# Optical coherence tomography angiography indicates subclinical retinal disease in neuromyelitis optica spectrum disorders

**DOI:** 10.1177/13524585211028831

**Published:** 2021-07-14

**Authors:** Lilian Aly, Eva-Maria Strauß, Nikolaus Feucht, Isabella Weiß, Achim Berthele, Meike Mitsdoerffer, Christian Haass, Bernhard Hemmer, Mathias Maier, Thomas Korn, Benjamin Knier

**Affiliations:** Department of Neurology, Klinikum rechts der Isar, Technical University of Munich, Munich, Germany/Institute for Experimental Neuroimmunology, Technical University of Munich, Munich, Germany; Department of Neurology, Klinikum rechts der Isar, Technical University of Munich, Munich, Germany/Institute for Experimental Neuroimmunology, Technical University of Munich, Munich, Germany; Department of Ophthalmology, Klinikum rechts der Isar, Technical University of Munich, Munich, Germany/Airport Munich Eyeclinic MVZ, Munich, Germany; Department of Ophthalmology, Klinikum rechts der Isar, Technical University of Munich, Munich, Germany; Department of Neurology, Klinikum rechts der Isar, Technical University of Munich, Munich, Germany; Department of Neurology, Klinikum rechts der Isar, Technical University of Munich, Munich, Germany/Institute for Experimental Neuroimmunology, Technical University of Munich, Munich, Germany; German Center for Neurodegenerative Diseases (DZNE), Munich, Germany/Institute of Metabolic Biochemistry, Biomedical Center (BMC), Faculty of Medicine, Ludwig-Maximilians-Universität München, Munich, Germany/Munich Cluster for Systems Neurology (SyNergy), Munich, Germany; Department of Neurology, Klinikum rechts der Isar, Technical University of Munich, Munich, Germany/Munich Cluster for Systems Neurology (SyNergy), Munich, Germany; Department of Ophthalmology, Klinikum rechts der Isar, Technical University of Munich, Munich, Germany; Department of Neurology, Klinikum rechts der Isar, Technical University of Munich, Munich, Germany/Institute for Experimental Neuroimmunology, Technical University of Munich, Munich, Germany/Munich Cluster for Systems Neurology (SyNergy), Munich, Germany; Department of Neurology, Klinikum rechts der Isar, Technical University of Munich, Munich, Germany/Institute for Experimental Neuroimmunology, Technical University of Munich, Munich, Germany

**Keywords:** Neuromyelitis optica spectrum disorders, optical coherence tomography angiography, astrocytes, disease activity, biomarker

## Abstract

**Background:**

Neuromyelitis optica spectrum disorders (NMOSD) are neuroinflammatory diseases of the central nervous system. Patients suffer from recurring relapses and it is unclear whether relapse-independent disease activity occurs and whether this is of clinical relevance.

**Objective:**

To detect disease-specific alterations of the retinal vasculature that reflect disease activity during NMOSD.

**Methods:**

Cross-sectional analysis of 16 patients with NMOSD, 21 patients with relapsing-remitting multiple sclerosis, and 21 healthy controls using retinal optical coherence tomography (OCT), optical coherence tomography angiography (OCT-A), measurement of glial fibrillary acidic protein (GFAP) serum levels, and assessment of visual acuity.

**Results:**

Patients with NMOSD but not multiple sclerosis revealed lower foveal thickness (FT) (*p* = 0.02) measures and an increase of the foveal avascular zone (FAZ) (*p* = 0.02) compared to healthy controls independent to optic neuritis. Reduced FT (*p* = 0.01), enlarged FAZ areas (*p* = 0.0001), and vessel loss of the superficial vascular complex (*p* = 0.01) were linked to higher serum GFAP levels and superficial vessel loss was associated with worse visual performance in patients with NMOSD irrespective of optic neuritis.

**Conclusion:**

Subclinical parafoveal retinal vessel loss might occur during NMOSD and might be linked to astrocyte damage and poor visual performance. OCT-A may be a tool to study subclinical disease activity during NMOSD.

## Introduction

Neuromyelitis optica spectrum disorders (NMOSD) are autoimmune diseases of the central nervous system (CNS) that mainly affect the optic nerve and the spinal cord. In the majority of cases, the pathology depends on the damage of astrocytes mediated by an auto-antibody to aquaporin-4 (AQP-4). In 10%–40% of NMOSD patients, antibodies to myelin oligodendrocyte glycoprotein (MOG) are detected, and anti-MOG antibody positive cases may be part of a distinct disease entity called MOG-antibody associated disease (MOGAD).^1^ Based on the current pathophysiological concept, neurological deficits in NMOSD are considered relapse-related. It is unclear whether silent, relapse-independent progression occurs and whether this might be clinically relevant.^1^ Optic neuritis (ON) is a core symptom of NMOSD causing vision loss due to severe neuroaxonal damage of both the optic nerve and the retina. Accordingly, retinal optical coherence tomography (OCT) reveals severe thinning of the retinal nerve fiber layer (RNFL) and the combined ganglion cell and inner plexiform layer (GCIP) in eyes that experienced ON.^2^

Based on recent observations, however, eyes of NMOSD patients also reveal retinal alterations with foveal thinning,^2^ flattening,^3,4^ and loss of parafoveal ganglion cells^5^ independently of previous ON. Furthermore, longitudinal neuroaxonal damage is apparent during NMOSD irrespective of ON attacks, suggesting that silent disease activity and progression might occur.^6^

Optical coherence tomography angiography (OCT-A) is a novel technique allowing rapid, non-invasive, and high-resolution imaging of the retinal vasculature. Recent studies have shown that retinal vessel loss occurs during relapsing-remitting multiple sclerosis (RRMS)^7,8^ and might also be found in individuals with NMOSD.^9^ In the current study, we used OCT-A to search for disease-specific alterations of the retinal vasculature in individuals with NMOSD, RRMS, and healthy controls (HC) and to test for their association with disease activity, disability, and CNS tissue damage.

## Material and methods

### Study design

In this cross-sectional analysis, we recruited individuals with NMOSD from the Department of Neurology, Klinikum rechts der Isar at the Technical University of Munich between 2018 and 2019. We included persons with RRMS and HC as controls. All individuals with NMOSD and RRMS also served as participants on an ongoing observational cohort study (TUM-MS cohort) with standardized annual assessments of the disease course, disease phenotype, and prior medical history. For definition of NMOSD and RRMS, we applied the 2015 international consensus criteria^10^ and the 2017 McDonald criteria,^11^ respectively. We excluded patients with substantial eye disease that may affect the integrity of the retinal architecture or vasculature (macular degeneration, retinal tumor, retinal detachment, vascular occlusions, history of eye surgery), refractory errors > 6 diopters (internal standard), or a relapse within 90 days before study enrollment. All individuals with NMOSD were tested for the presence of both serum antibodies against AQP-4 and MOG using a commercially available cell-based assay (Euroimmun, Germany). Individuals with MOGAD were excluded from the study. All patients underwent retinal OCT and OCT-A examination. A detailed medical history including chart review especially on former ON history was taken from all individuals. Disease duration was calculated as the time period between first symptom onset and enrollment. Patients and controls received a thorough physical examination and assessment of the Expanded Disability Status Scale (EDSS). We assumed a history of subclinical ON in individuals with intereye differences of both the peripapillary RNFL (pRNFL) and the GCIP of more than 5 and 4 µm as detected by OCT, respectively.^12^ In case of suspected subclinical ON, eyes were treated as eyes with a history of clinical ON. We measured monocular high-contrast visual activity (HCVA, 100%) and low-contrast visual acuity (LCVA, 2.5%) using Early Treatment Diabetic Retinopathy Study charts placed in a retro-illuminated cabinet with best refractive correction. Venous blood was taken and serum was stored at −80°C in the biobank of the Department of Neurology for additional analysis of fluid biomarkers. The study was approved by the ethics commission of the Technical University of Munich, School of Medicine, and adhered to the Declaration of Helsinki. All participants gave written informed consent.

### Retinal imaging

OCT (Heidelberg Engineering Spectralis OCT) images were acquired as described elsewhere^13^ and included examination of the pRNFL (12° ring scan) and the macula (30° × 25° macular scan). We checked all scans for sufficient quality according to the OSCAR-IB criteria.^14^ Retinal segmentation of the GCIP and the inner nuclear layer (INL) was performed automatically by an inbuilt software algorithm (Eye Explorer, v2.5.4.) and manually corrected if necessary. Foveal thickness (FT) was measured as the mean thickness of a 1-mm-diameter cylinder around the fovea.^2^ OCT-A image acquisition was performed using a RTVue XR Avanti OCT (OptoVue Inc.) as previously described.^7^ In brief, we assessed en face images and decorrelation signals representing vessel densities of a 6 mm × 6 mm area focusing on the fovea centralis. Segmentation of the superficial (SVC) and deep vascular complex (DVC) within a circle around the fovea between 1 and 3 mm eccentricity and assessment of the foveal avascular zone (FAZ) was performed automatically. To ensure sufficient OCT-A image quality, we only included examinations with a signal strength index of ⩾60 and correct segmentation. Images with obvious problems, decentration of the imaging focus, and major motion artifacts defined as a motion artifact score^15^ > 2 were excluded.

### Serum analysis

Serum neurofilament light chain (sNfL) and serum glial fibrillary acidic protein (sGFAP) were measured using Simoa assays^16–18^ (Quanterix; NF light Simoa Assay Advantage Kit, GFAP Simoa Discovery Kit). All samples were analyzed for the same target (sNFL or sGFAP) at the same time point. Samples were thawed and processed as recommended by the manufacturer and previously described.^19^ We assessed the intra-assay coefficient of variation (CV) by testing a quality control serum sample in five replicates. A CV of lower than 10% had to be achieved for valid analysis. Concentrations were calculated using corresponding standard curves. The laboratory personnel were blinded for clinical data.

### Data availability

Data are available upon reasonable request. We will share raw imaging OCT-A data in an anonymized way upon request by any qualified investigator. The data are not publicly available due to privacy or ethical restrictions.

### Statistical analysis

We applied GraphPad Prism (v9.1.0). To account for intereye correlations, we used a paired-eye statistical approach.^13^ Mean values of both eyes were used as one data point when both eyes were available and allocated to the same group (ON, no ON). If one eye was excluded, values of the remaining eye were used. To evaluate cross-sectional differences between groups, we performed an ordinary one-way analysis of variance (ANOVA) with Tukey’s multiple comparisons or a non-parametric Kruskal–Wallis test with Dunn’s multiple comparisons. Differences between two groups were analyzed using Student’s *t*-test. Multiple linear regression models were applied to test the impact of OCT or OCT-A values on disease patterns, soluble biomarkers, and visual acuity. We corrected all models for the covariates age and sex if not otherwise stated and provide the respective estimates (β-value) as regression parameters. Values are provided as mean ± standard deviation if normally distributed, otherwise as median (25%–75% interquartile range). The statistical significance threshold was *p* < 0.05.

## Results

### Study cohort

We included 16 individuals with NMOSD, 21 with RRMS, and 21 HCs. Two eyes of two NMOSD patients were excluded due to poor OCT-A quality. One RRMS patient had undergone unilateral enucleation in the past. Ten patients (63%) with NMOSD had serum antibodies to AQP-4 and 6 patients were negative for both serum antibodies against AQP-4 and MOG. Patients with NMOSD revealed higher EDSS values as compared to individuals with RRMS whereas age and sex were comparable (Table 1). Both patients with NMOSD and RRMS had lower visual acuities than HC individuals whereas we did not see any differences between patients with NMOSD or RRMS (Table 1).

**Table 1.** Study population.

	HC (*n* = 21; 42 eyes)	MS (*n* = 21; 41 eyes)	NMOSD (*n* = 16; 30 eyes)	*p*-value
Female, no. (%)	16 (76)	16 (76)	13 (81)	0.92
Age, years	42.0 ± 9.5	38.0 ± 11.4	46.6 ± 10.0	0.06
Disease duration, months	n.a.	68 ± 49	73 ± 31	0.67
EDSS score	n.a.	1.4 ± 1.2	3.4 ± 2.4	**0.0009**
Immunotherapy no. (%)	n.a.	19 (90)	16 (100)	0.50
Alemtuzumab		1 (5)	0 (0)	
Azathioprine		0 (0)	2 (13)	
Dimethyl fumarate		4 (19)	0 (0)	
Eculizumab		0 (0)	1 (6)	
Fingolimod		3 (14)	0 (0)	
Glatiramer acetate		2 (10)	1 (6)	
Interferon beta		3 (14)	0 (0)	
Natalizumab		3 (14)	0 (0)	
Ocrelizumab/rituximab		4 (19)	10 (63)	
Tocilizumab		0 (0)	2 (13)	
History of one-sided clinical ON, no. (%)	n.a.	10 (48)	8 (50)	0.75
Prior one-sided subclinical ON, no. (%)	0 (0)	2 (10)	0 (0)	>0.99
History of both-sided clinical ON, no. (%)	n.a.	2 (10)	1 (6)	>0.99
HCVA, no ON	1.4 ± 0.3	1.0 ± 0.3	1.1 ± 0.3	**0.0002** ^a^
HCVA, ON	n.a.	0.8 ± 0.3	1.0 ± 0.4	0.22
LCVA, no ON	0.4 ± 0.1	0.2 ± 0.1	0.3 ± 0.1	**0.0005** ^b^
LCVA, ON	n.a.	0.2 ± 0.1	0.3 ± 0.2	0.17

HC: healthy controls; MS: multiple sclerosis; NMOSD: neuromyelitis optica spectrum disorders; EDSS: Expanded Disability Status Scale; ON: optic neuritis; HCVA: high-contrast visual acuity; LCVA: low-contrast visual acuity; n.a.: not available.

Demographics of healthy controls (HC) and individuals with relapsing-remitting MS (MS) or neuromyelitis optica spectrum disorders (NMOSD); HCVA, LCVA in eyes without (no ON) or with a history of previous optic neuritis (ON). Bold values indicate a significance level of *p*<0.05.

a HC versus MS *p* = 0.0001, HC versus NMOSD *p* = 0.01.

b HC versus MS *p* = 0.0004, HC versus NMOSD *p* = 0.02.

### OCT analysis in patients with NMOSD and RRMS

As expected and in line with the literature,^1,20,21^ eyes in both patients with RRMS and NMOSD that had suffered from ON in the past revealed lower pRNFL and GCIP measures as compared to HC eyes or eyes without an ON history (Table 2). Notably, we found a significant reduction of the FT in patients with NMOSD but not RRMS as compared to HC irrespectively of an ON history (Table 2).

**Table 2.** Results of optical coherence tomography and optical coherence tomography angiography analysis.

	HC (*n* = 21)	MS (*n* = 21)		NMOSD (*n* = 16)		*p*-value
	No ON (42 eyes)	No ON (26 eyes)	ON (15 eyes)	No ON (21 eyes)	ON (9 eyes)	
pRNFL, µm	103 ± 7	99 ± 11	88 ± 12	98 ± 18	72 ± 19	**<0.0001** ^a^
GCIP, mm^3^	2.0 ± 0.1	1.9 ± 0.2	1.7 ± 0.2	1.9 ± 0.2	1.5 ± 0.4	**<0.0001** ^b^
INL, mm^3^	1.1 ± 0.1	1.1 ± 0.1	1.1 ± 0.1	1.1 ± 0.1	1.1 ± 0.1	0.16
FT, µm	282 ± 17	279 ± 22	268 ± 23	261 ± 17	253 ± 15	**0.002** ^c^
SVC, % vessel density	53.3 ± 2.5	51.8 ± 2.6	50.4 ± 3.7	51.0 ± 3.8	47.4 ± 4.3	**0.0008** ^d^
DVC, % vessel density	57.3 ± 5.5	57.2 ± 5.7	59.1 ± 3.9	56.9 ± 5.1	57.0 ± 3.9	0.76
FAZ, mm^2^	0.20 ± 0.07	0.22 ± 0.10	0.28 ± 0.14	0.29 ± 0.09	0.32 ± 0.09	**0.005** ^e^

HC: healthy controls; MS: multiple sclerosis; NMOSD: neuromyelitis optica spectrum disorders; ON: optic neuritis; pRNFL: peripapillary retinal nerve fiber layer; GCIP: combined ganglion cell and inner plexiform layer; INL: inner nuclear layer; FT: foveal thickness; SVC: superficial vascular complex; DVC: deep vascular complex; FAZ: foveal avascular zone.

Results from optical coherence tomography (OCT) and OCT angiography analysis in healthy controls (HC) and individuals with relapsing-remitting MS (MS) or neuromyelitis optica spectrum disorders (NMOSD) in eyes without (no ON) or with a history of optic neuritis (ON). pRNFL, GCIP, INL, and FT as measured by OCT. Macular SVC, DVC, and FAZ as measured by OCT angiography. Bold values indicate a significance level of *p*<0.05.

a HC versus MS-ON *p* = 0.008, HC versus NMOSD-ON *p* = 0.0002, NMOSD-no ON versus NMOSD-ON *p* = 0.006.

b HC versus MS-ON *p* = 0.002, HC versus NMOSD-ON *p* < 0.0001, NMOSD-no ON versus NMOSD-ON *p* = 0.002.

c HC versus NMOSD-no ON *p* = 0.02, HC versus NMOSD-ON *p* = 0.003.

d HC versus NMOSD-ON *p* = 0.0004, MS-no ON versus NMOSD-ON *p* = 0.02.

e HC versus NMOSD-ON *p* = 0.04.

### OCT and OCT-A findings in NMOSD and RRMS

**Figure 1.**
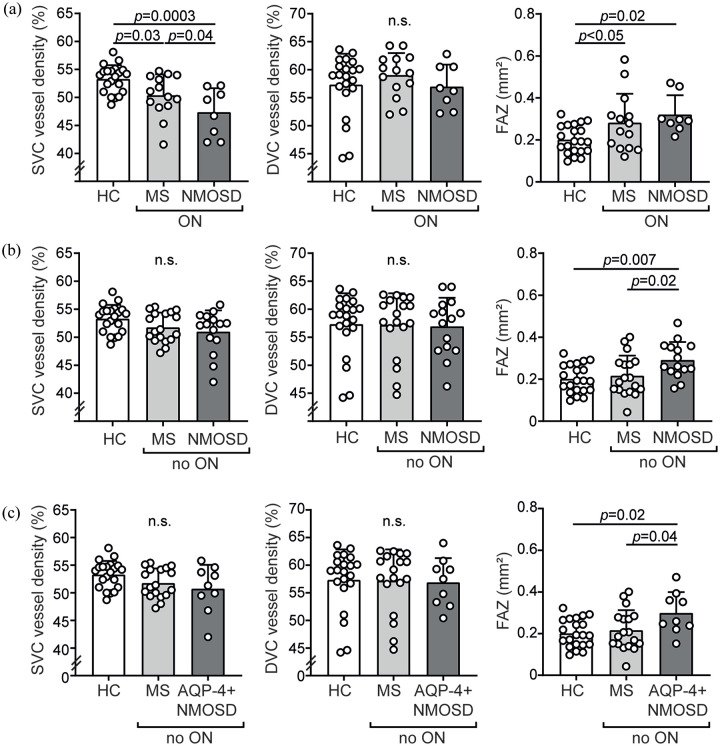
Results of optical coherence tomography angiography analysis. (a) Parafoveal vessel densities of the superficial (SVC) and deep vascular complex (DVC) and size of the foveal avascular zone (FAZ) in healthy individuals (HC, *n* = 21) and eyes with former optic neuritis (ON) of patients with relapsing-remitting MS (MS, *n* = 14 patients) and neuromyelitis optica spectrum disorders (NMOSD, *n* = 8 patients); one eye of one NMOSD patient was excluded due to poor OCT quality. (b) Parafoveal vessel densities of the SVC and DVC and size of the FAZ in HC (*n* = 21), MS (*n* = 18 patients), and NMOSD (*n* = 15 patients) in eyes without a history of optic neuritis (no ON); one MS patient underwent unilateral enucleation in the past. (c) Parafoveal vessel densities of the SVC and DVC and size of the FAZ in HC (*n* = 21), MS (*n* = 18 patients), and NMOSD with antibodies against aquaporin-4 (AQP-4 + NMOSD) (*n* = 9 patients) in eyes without a history of optic neuritis (no ON). (a–c) Mean ± standard deviation (SD); symbols depict single patient values; one-way ANOVA; n.s.: not significant; the statistical significance threshold was *p*<0.05.

Considering vascular alterations as detected by OCT-A, eyes of individuals with RRMS and NMOSD and a history of ON revealed lower vessel densities of the SVC and increased FAZ measures as compared to eyes of HC (Figure 1(a); Table 2). We did not recognize any vascular differences between ON eyes of individuals with RRMS and NMOSD (Table 2). In eyes without former ON (no ON), patients with NMOSD, but not RRMS, revealed an increase in the FAZ as compared to HC whereas we did not see any differences in the SVC and DVC densities between individuals with RRMS and NMOSD (Figure 1(b); Table 2). The results remained robust when only considering NMOSD with anti-AQP-4 antibodies (Figure 1(c)).

When combining all eyes without an ON history from all groups, lower vessel densities of the DVC were linked to a higher age (β = −0.26, 95% confidence interval (CI) = −0.36 to −0.14, *p* < 0.0001; multiple linear regression analysis corrected for sex). We did not see any associations of the remaining OCT-A measures with age or sex (data not shown). Vessel densities of the SVC correlated positively with pRNFL thicknesses (β = 0.10, 95% CI = 0.04 to 0.16, *p* = 0.0008). The FAZ area correlated negatively with FT measures (β = −0.003, 95% CI = −0.004 to −0.002, *p* < 0.0001).

### Association of retinal pathology, surrogate markers of CNS tissue damage, and visual function

**Figure 2.**
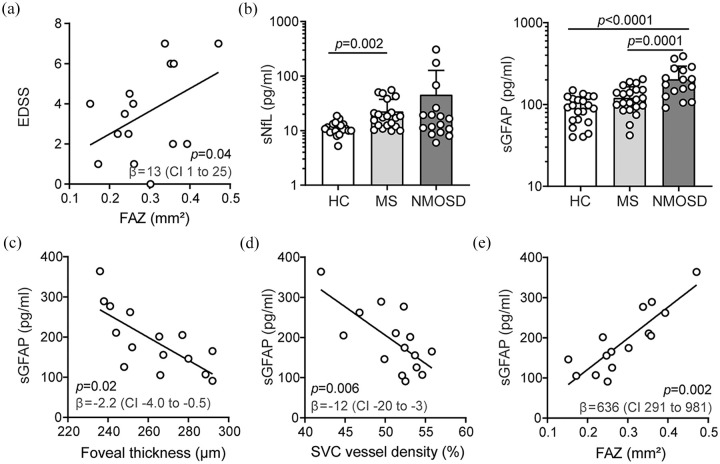
Retinal architecture, vasculature, and surrogate markers of disease activity in neuromyelitis optica spectrum disorders. (a) Association of the Expanded Disability Status Scale (EDSS) and the foveal avascular zone (FAZ) areas during neuromyelitis optica spectrum disorders (NMOSD) (*n* = 15); β regression estimate and 95% confidence interval (CI); symbols depict single patients; multiple linear regression model corrected for age and sex. (b) Serum levels of neurofilament light chain (sNfL) and glial fibrillary acidic protein (sGFAP) in healthy individuals (HC, *n* = 21), patients with relapsing-remitting MS (MS, *n* = 21) and NMOSD (*n* = 15); mean ± standard deviation; symbols depict single patients; Kruskal–Wallis test (sNfL), one-way ANOVA (GFAP). (c–e) Association of sGFAP levels and foveal thickness (FT) (c), vessel densities of the superficial vascular complex (SVC) (d), and the foveal avascular zone (FAZ) areas (e) in eyes without former optic neuritis in patients with NMOSD (*n* = 15); β regression estimates and 95% CI; symbols depict single patients; multiple linear regression models corrected for age, sex, and EDSS.

In the next step, we tested whether alterations of the retinal architecture and vasculature were associated with established disease activity markers. As described earlier (Table 1), patients with NMOSD had higher EDSS values compared to RRMS. In NMOSD, EDSS correlated with FAZ areas (Figure 2(a)) and GCIP volumes (β = −4.4, 95% CI = −8.4 to −0.3, *p* = 0.04) in no ON eyes.

Recently, sNfL and sGFAP have been introduced as new biomarkers for disease activity and disability in RRMS^16,17^ and NMOSD.^18,22^ In our cohort, sNfL was elevated in RRMS and NMOSD as compared to HC (Figure 2(b)). As expected,^16^ higher sNfL measures were associated with higher EDSS values (β = 7.5, 95% CI = 0.1 to 14.8, *p* < 0.05) in patients with RRMS but not NMOSD. We did not find any robust associations of OCT or OCT-A measures and sNfL levels in either NMOSD or RRMS patients (data not shown).

Focusing on sGFAP, NMOSD patients showed higher levels as compared to RRMS patients and HC and no clear differences were observed between RRMS and HC (Figure 2(b)). Serum levels of GFAP were linked to higher EDSS measures in NMOSD (β = 28, 95% CI = 9 to 47, *p* = 0.008). When correcting for age, sex, and EDSS, an ON-independent foveal thinning was linked to higher sGFAP levels in patients with NMOSD but not MS or HC (Figure 2(c)). In a comparable way, lower SVC vessel densities (Figure 2(d)) and larger FAZ areas (Figure 2(e)) were associated with increased sGFAP levels when considering no ON eyes in NMOSD but not RRMS. When including SVC vessel density and FT as additional covariates next to age, sex, and EDSS, FAZ areas remained linked to sGFAP levels in individuals with NMOSD (β = 520, 95% CI = 36 to 1004, *p* = 0.04; multiple linear regression analysis, no ON eyes only). We found no associations of sGFAP levels with clinical patterns, OCT, and OCT-A measures in patients with RRMS.

**Figure 3.**
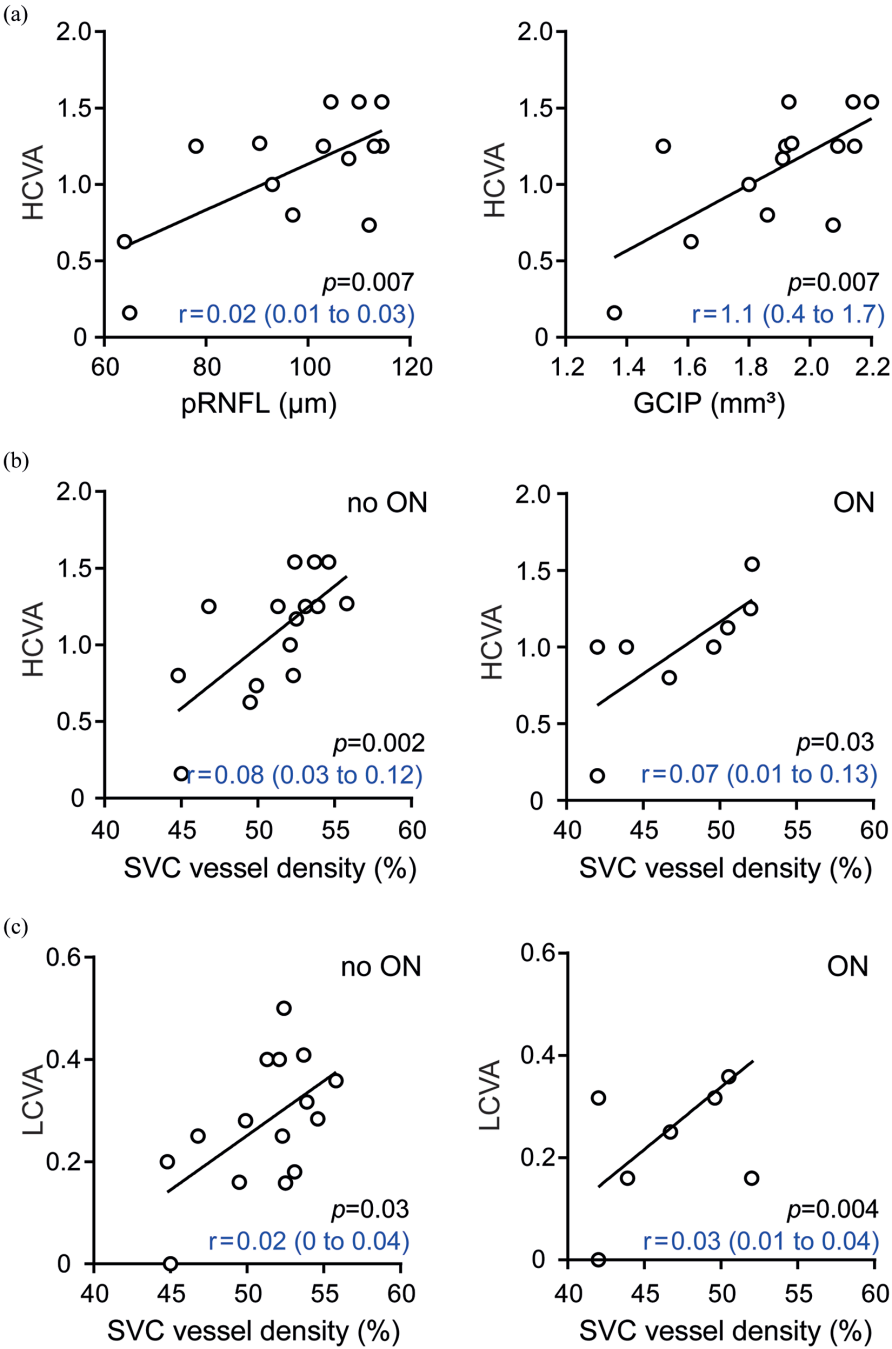
Retinal architecture, vasculature, and visual function in neuromyelitis optica spectrum disorders. (a) Association of high-contrast visual acuity (HCVA) and thickness of the peripapillary retinal nerve fiber layer (pRNFL, left) or volumes of the common ganglion cell and inner plexiform layer (GCIP, right) in eyes without former optic neuritis (ON) in patients with neuromyelitis optica spectrum disorders (NMOSD) (*n* = 14). (b) Association of HCVA and vessel densities of the superficial vascular complex (SVC) in eyes of NMOSD patients without (*n* = 15, left) or with a history of optic neuritis (*n* = 8, right). (c) Association of low-contrast visual acuity (LCVA) and vessel densities of SVC in eyes of NMOSD patients without (*n* = 15, left) or with a history of optic neuritis (*n* = 8, right). (a–c) β regression estimates and 95% CI; symbols depict single patients; multiple linear regression model corrected for age and sex.

Finally, we assessed functional visual tests in the context of altered retinal architectures of individuals with NMOSD. HCVA was linked to pRNFL thickness in eyes without ON (Figure 3(a)) and GCIP volumes in both eyes with (β = 1.0, 95% CI = 0.3 to 1.6, *p* = 0.01) and without ON (Figure 3(a)). As expected,^23^ we found an association of LCVA values with pRNFL (β = 0.01, 95% CI = 0 to 0.02, *p* = 0.05) and GCIP measures (β = 0.3, 95% CI = 0.1 to 0.6, *p* = 0.02) in ON eyes. Furthermore, reduced SVC vessel densities in NMOSD eyes were linked to an impaired visual function as measured by HCVA (Figure 3(b)) and LCVA (Figure 3(c)) irrespective of any ON history. We did not find associations between FAZ, DVC, and visual function in patients with NMOSD in eyes either with or without ON. No associations of the retinal vasculature and visual functions were found in patients with RRMS.

## Discussion

Our data indicate subclinical and relapse-independent alterations of the retinal architecture and vasculature during NMOSD. Here, ON-independent vessel loss of the SVC especially in parafoveal areas might occur during NMOSD, which might be linked to a loss of astrocytes and poor visual performance. These data suggest that OCT-A might be a cost-effective tool to study subclinical disease activity and astrocyte loss in individuals with NMOSD.

As reported previously, we confirm that superficial retinal vessel loss occurs during NMOSD.^9,24,25^ We found comparable alterations of the SVC and DVC in RRMS and NMOSD. Parafoveal ON-independent vessel loss resulting in increased FAZ areas, however, was found as a unique feature in NMOSD eyes that might allow discrimination from RRMS. There are only few studies comparing retinal vascular alteration between individuals with RRMS and NMOSD. Lee et al.^24^ found a pronounced vessel loss within the SVC in patients with NMOSD as compared to RRMS in eyes with ON that might allow discrimination of both disease entities. In this article, however, FAZ areas and eyes without ON have not been addressed. A recent study from Rogaczewska et al.^26^ described a more severe vessel loss in peripapillary areas occurring after ON in NMOSD as compared to RRMS whereas macular areas have not been analyzed. As a consequence, further studies are needed to confirm our findings and to assess the discriminatory potential of the FAZ to distinguish NMOSD from RRMS in eyes without a history of ON.

As already shown by other groups,^2–4^ we found foveal thinning as a unique feature in patients with NMOSD but not RRMS and HC irrespective of ON. In line with this, individuals with NMOSD also revealed enlargement of the FAZ suggesting loss of the parafoveal vasculature. This phenomenon might be directly linked to the pathology of AQP-4 antibody-positive NMOSD since parafoveal areas of the retina contain the highest density of astrocytic Müller cells,^27^ which express AQP-4 and have shown to be targets of anti-AQP-4 antibodies in NMOSD.^2^ Here, atrophy of the fovea and loss of the parafoveal vasculature occur independently of ON. This suggests that subclinical disease activity occurs in NMOSD and might drive relapse-independent disease progression. Foveal thinning and parafoveal vessel loss could be a surrogate of this process in NMOSD. This hypothesis is backed by the observation that parafoveal retinal vessel loss was linked to increased sGFAP levels in NMOSD patients in our study. sGFAP is very likely derived from damaged astrocytes. A recent publication showed that sGFAP measures increase after relapses and may accumulate due to tissue damage correlating with disability.^22^ Furthermore, there is novel and convincing data that sGFAP levels are associated with relapse frequency, relapse severity, and treatment efficacy in individuals with NMOSD suggesting that sGFAP measures could serve as a biomarker for this disease entity.^18^ Given the possible linkage of foveal thinning, parafoveal vessel loss, and markers for astrocytic decline during NMOSD, the data of others^2–4^ and us can help to establish the hypothesis that a damage of the blood-retinal barrier due to loss of Müller cells might cause the rarefication of retinal vessels and an increase of the FAZ in NMOSD but not RRMS.

Besides the association of retinal vascular alterations and markers for CNS damage during NMOSD, we found an association of retinal vessel loss and worse visual performance in NMOSD but not RRMS. Here, relapse-independent vessel loss and enlargement of the FAZ might have relevant clinical impact since both features are associated with poor visual performance. Integrity of visual function has furthermore been shown to strongly affect quality of life.^23^ If confirmed by other groups, this observation might affect clinical decision making in the future. Currently, therapeutic decision making in NMOSD relies on relapse frequency and severity. After introduction of novel and NMOSD-specific therapeutic interventions,^28^ assessment of subclinical disease activity and the phenomenon of silent worsening of visual function should be included in the design of therapeutic strategies for NMOSD.

Our study has several limitations: first, we did not assess the longitudinal time course of retinal vessel loss, sGFAP levels, and its association with visual performance. Here, future longitudinal studies are needed. OCT-A, however, is a novel technique and longitudinal data on retinal vessel alterations are limited at the moment. Second, additional subgroup analyses in NMOSD patients depending on the presence or absence of anti-AQP-4 antibodies were not performed due to the moderate sample size. Yet, we could show that our findings remained robust when only considering patients with anti-AQP-4 antibodies. NMOSD, however, is a very rare disease entity and further multi-center studies enabling bigger cohorts might provide further insights into NMOSD subtypes as well as patients with MOGAD. Third, we did not perform a standardized magnetic resonance imaging (MRI) analysis of the brain and especially the optic nerves in our patients with NMOSD. ON during NMOSD can involve the optic chiasm and thus a clinical unilateral ON might also affect the contralateral eye. Here, we cannot exclude a possible influence of ON episodes on the vasculature of the contralateral eye. Fourth, we can only speculate whether retinal Müller cells or spinal cord or brain astrocytes are the major source of sGFAP in relapse-free patients with NMOSD. However, since we found an association of sGFAP, visual performance, and parafoveal vessel densities, retinal Müller cells are very likely affected during NMOSD. Here, additional preclinical data from experimental models are needed to confirm this hypothesis.

Taken together, out study suggests that subclinical parafoveal vessel loss occurs as a unique feature during NMOSD but not RRMS. Vessel loss and enlargement of the FAZ might be linked to NMOSD disease activity, astrocyte loss, and visual performance. Thus, retinal OCT-A may be a convenient tool to study subclinical disease activity in NMOSD.
